# Down-regulation of IKKβ expression in glioma-infiltrating microglia/macrophages is associated with defective inflammatory/immune gene responses in glioblastoma

**DOI:** 10.18632/oncotarget.5310

**Published:** 2015-09-29

**Authors:** Jakub Mieczkowski, Marta Kocyk, Pawel Nauman, Konrad Gabrusiewicz, Małgorzata Sielska, Piotr Przanowski, Marta Maleszewska, Wenson D. Rajan, Dominika Pszczolkowska, Tomasz Tykocki, Wieslawa Grajkowska, Katarzyna Kotulska, Marcin Roszkowski, Boguslaw Kostkiewicz, Bozena Kaminska

**Affiliations:** ^1^ Laboratory of Molecular Neurobiology, Nencki Institute of Experimental Biology, Warsaw, Poland; ^2^ Postgraduate School of Molecular Medicine, Medical University of Warsaw, Poland; ^3^ Department of Neurosurgery, Institute Psychiatry and Neurology, Warsaw, Poland; ^4^ Departments of Pathology, The Children's Memorial Health Institute, Warsaw, Poland; ^5^ Neurology, The Children's Memorial Health Institute, Warsaw, Poland; ^6^ Neurosurgery, The Children's Memorial Health Institute, Warsaw, Poland; ^7^ Central Clinical Hospital Ministry of Interior, Warsaw, Poland

**Keywords:** immunosuppression, glioma-associated microglia/macrophages, global gene expression profiling, glioblastoma, IKKβ/NFkB signaling

## Abstract

Glioblastoma (GBM) is an aggressive malignancy associated with profound host immunosuppression. Microglia and macrophages infiltrating GBM acquire the pro-tumorigenic, M2 phenotype and support tumor invasion, proliferation, survival, angiogenesis and block immune responses both locally and systematically. Mechanisms responsible for immunological deficits in GBM patients are poorly understood. We analyzed immune/inflammatory gene expression in five datasets of low and high grade gliomas, and performed Gene Ontology and signaling pathway analyses to identify defective transcriptional responses. The expression of many immune/inflammatory response and TLR signaling pathway genes was reduced in high grade gliomas compared to low grade gliomas. In particular, we found the reduced expression of the *IKBKB,* a gene coding for IKKβ, which phosphorylates IκB proteins and represents a convergence point for most signal transduction pathways leading to NFκB activation. The reduced *IKBKB* expression and IKKβ levels in GBM tissues were demonstrated by qPCR, Western blotting and immunohistochemistry. The IKKβ expression was down-regulated in microglia/macrophages infiltrating glioblastoma. NFκB activation, prominent in microglia/macrophages infiltrating low grade gliomas, was reduced in microglia/macrophages in glioblastoma tissues. Down-regulation of *IKBKB* expression and NFκB signaling in microglia/macrophages infiltrating glioblastoma correlates with defective expression of immune/inflammatory genes and M2 polarization that may result in the global impairment of anti-tumor immune responses in glioblastoma.

## INTRODUCTION

Gliomas are the common primary tumors in the central nervous system. According to the World Health Organization gliomas are divided into four histological grades (WHO I-IV), tightly coupled to patient outcome. Type and quality of immune responses in tumor microenvironment are predictive of patient outcome [1]. Aggressive gliomas progress despite high infiltration by immune cells [2–4]. Immune responses in high grade gliomas (III-IV) are characterized by low peripheral lymphocyte counts, impaired mitogen-induced responses of peripheral mononuclear cells, accumulation of CD8^+^ suppressor T cells and CD4^+^CD25^+^FoxP3^+^Treg cells [3, 5, 6]. Adaptive immune responses are deficient, with diminished responsiveness of peripheral T cells and reduced induction of immunoglobulin synthesis by B cells [7, 8].

Histopathology and flow cytometry studies have shown accumulation of microglia, blood-derived macrophages and myeloid-derived suppressor cells (MDSC) in human gliomas [9–13]. Infiltrating microglia/macrophages do not secrete pro-inflammatory cytokines, have impaired cytotoxicity, and stimulate the accumulation of T-suppressor cells [5, 6, 14, 15]. Tumor-associated macrophages can adapt M1 or M2 phenotype and exert anti-tumoral and pro-tumoral functions, respectively [16, 17]. Glioma-associated microglia/macrophages (due to a lack of reliable immunohistochemistry marker collectively called “microglia/macrophages” or GAMs) display features of M2 phenotype and express many factors, chemokines and cytokines that support tumor invasion and angiogenesis, and stimulate local and systemic immunosuppression in glioma patients [18] via mechanisms that still need to be defined. The pro-invasive activity of GAMs have been demonstrated in microglia-glioma co-cultures, in brain organotypic slice cultures and experimental murine gliomas [19–24]. Gene expression profiling of rodent GAMs and microglia exposed to glioma cells demonstrates the pro-invasive and immunosuppressive M2 phenotype of these cells [25, 26], with low or absent expression of many immune response genes [26].

Transcriptomic studies of glioblastoma (GBM) have identified different signatures associated with glioma pathogenesis, overall survival or response to treatment [27–30]. The analysis of allergy- and inflammation-related genes in GBM demonstrated an inverse correlation between their high expression and GBM aggressiveness. Co-expression network analysis of 791 immune-related genes associated with patient survival in GBM disclosed innate immune system, natural killer and myeloid cell signatures [31]. Increased expression of immune-related genes in high grade astrocytoma correlated with longer survival [32].

To search for molecular mechanisms of immunosuppression in human gliomas, we performed a meta-analysis of immune and inflammation-related gene expression in merged datasets of low- and high-grade gliomas. The reduced expression of many immune responses and TLR signaling genes, down-regulation of IKKβ and deficiency of NFκB activation was demonstrated in high grade gliomas. Defective IKKβ expression and NFκB activation in GAMs may result in defective expression of immune genes and anti-tumor immune responses in glioblastoma.

## RESULTS

### Microglia/macrophages accumulate and are activated both in low and high grade gliomas

**Figure 1:**
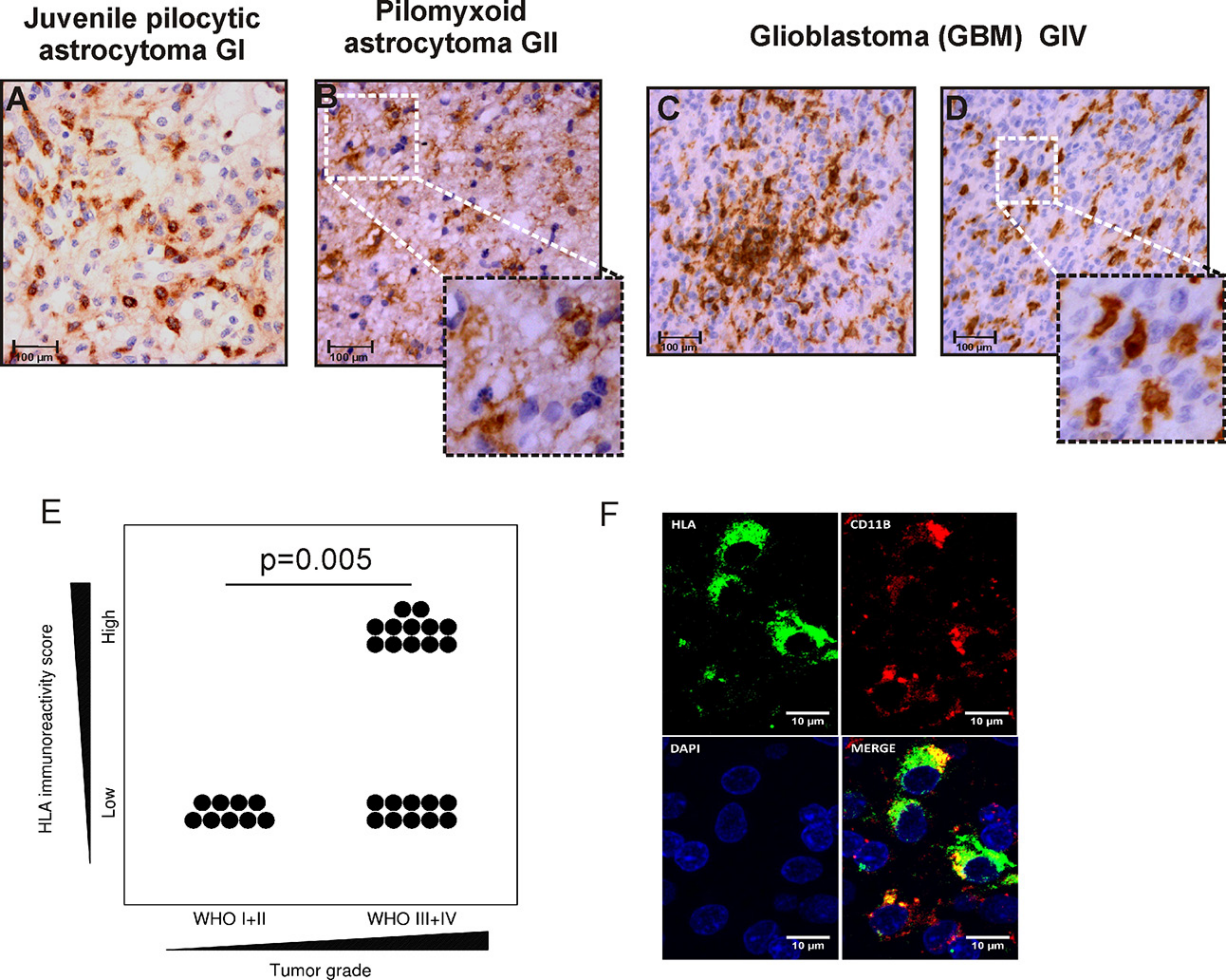
Detection of microglia/macrophages in low and high grade gliomas **A-D.** HLA-DP,-DQ,-DR (HLA) expression was determined with CR3/43 antibody by immmunohistochemistry on paraffin-embedded sections. Intense HLA staining (brown) is present intratumorally and around vessels. High magnification pictures show a representative morphology of the HLA immunoreactive cells in low grade astrocytomas and GBM (scale bars 100 μm, insets: 50 μm). **E.** Quantification of immunoreactivity in low and high grade gliomas among adult patients. F. Double staining for HLA-DP,-DQ,-DR and CD11b shows signal co-localization in a majority of the positive population.

In the presented study, abundance and distribution pattern of microglia/macrophages in low and high grade brain tumors was analyzed by immunocytochemistry with an anti-HLA CR3/43 antibody. We found strong HLA immunoreactivity representing the presence of microglia/macrophages in low and high grade gliomas. HLA-positive cells with ramified, hypertrophic morphology were detected more frequently in low grade tumors such as juvenile pilocytic astrocytoma (JPA) and pilomyxoid astrocytoma (Fig. 1A, 1B inset). Numerous amoeboid, HLA-positive cells were present in the parenchyma and around blood vessels in GBM (Fig. 1C, 1D). Tumor-infiltrating microglia/macrophages showed the stronger intensity of HLA staining than the immunoreactive cells in tissues surrounding the tumor mass. Interestingly, HLA-immunoreactive cells were not detected in non-glial tumors such as meningiomas WHO grade I (*n* = 3) and supratentorial primitive neuroectodermal tumors WHO grade IV (*n* = 4) (not shown). The HLA immunoreactivity score was significantly higher in GBM than in low grade gliomas in adults (*p* < 0.05) (Fig. 1E). Double staining with anti-CD11b and anti-HLA-CR3/43 antibodies demonstrates co-localization of both markers in a majority of positive cells (Fig. 1F).

### The expression of innate immune/immune/inflammatory genes is impaired in high grade gliomas

In order to study immune responses in low and high grade gliomas, we performed a meta-analysis of gene expression based on five publicly available datasets, merged in 60 profiles of low grade and 132 of high grade gliomas (supplementary Table 1 contains information on histopathological classification of tumors enclosed). The expression values were preprocessed using a previously described procedure [33] and aggregated. The boxplots present overall gene expression distributions in all samples after quantile normalization. The association between original data sets (before merging) is clearly visible (Supplementary Fig. 1).

**Figure 2:**
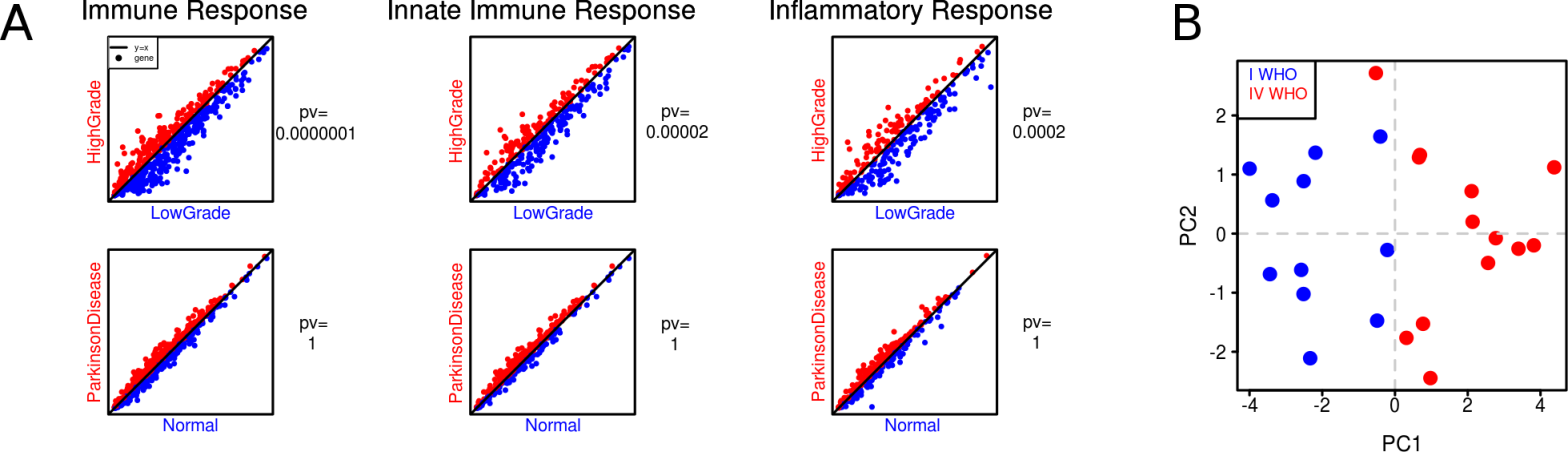
The expression of immune/innate immune/inflammatory response genes in low and high grade gliomas **A.** Visualization of averaged expression values: columns stand for GO terms in merged glioma datasets and an additional dataset (patients with Parkinson disease and healthy individuals). A single plot shows comparison between average gene expression values of each gene from a given GO term (low grade gliomas in blue, high grade ones in red). Coordinates of each gene are its averaged expression values in compared groups. The *p*-values were computed with a paired *t*-test. **B.** Principal Component Analysis (PCA) of immune response gene expression in low and high grade gliomas. Microarray analysis of an independent set of 11 JPA and 13 GBM was performed followed by PCA using a set of genes from the computed “immune” signature; each sample is plotted on the plane of the first two principal directions based on their expression (JPA in blue, GBM in red).

To evaluate expression profiles of the immune response genes, we used three Gene Ontology (GO) terms best describing immune responses namely: Immune Response, Innate Immune Response and Inflammatory Response (supplementary Table 2). A single plot shows comparison between average gene expression values for a given GO term (Fig. 2A). Coordinates of each gene reflect its averaged expression value in compared groups. The red color marks the higher expression in high-grade gliomas, while the blue color represents the higher expression in low-grade gliomas. The expression of the innate immune, immune and inflammatory genes was significantly higher in low grade than in high grade gliomas that suggests the effective anti-tumor responses operating in low grade gliomas. The similar analysis performed on the dataset consisting microarray expression profiles of normal and Parkinson brains showed the higher expression of innate immune, immune and inflammatory genes in brains of patients with Parkinson disease.

We performed Prediction Analysis of Microarrays (PAM) to select genes that belong to the three GO terms and best discriminate low grade from high grade gliomas. Using PAM we identified a minimal subset of genes which discriminated between groups. The ‘immune signature’ containing 41 genes was selected using a threshold of 5. The computed “immune” signature was then validated using an independent set of 11 JPA and 13 GBM. We performed Principal Component Analysis (PCA) on these samples using only genes that belong to the “immune” signature. Fig. 2B shows good discrimination between JPA and GBM achieved with the “immune” signature.

### The expression of TLR signaling pathway genes is decreased in high grade gliomas

**Figure 3:**
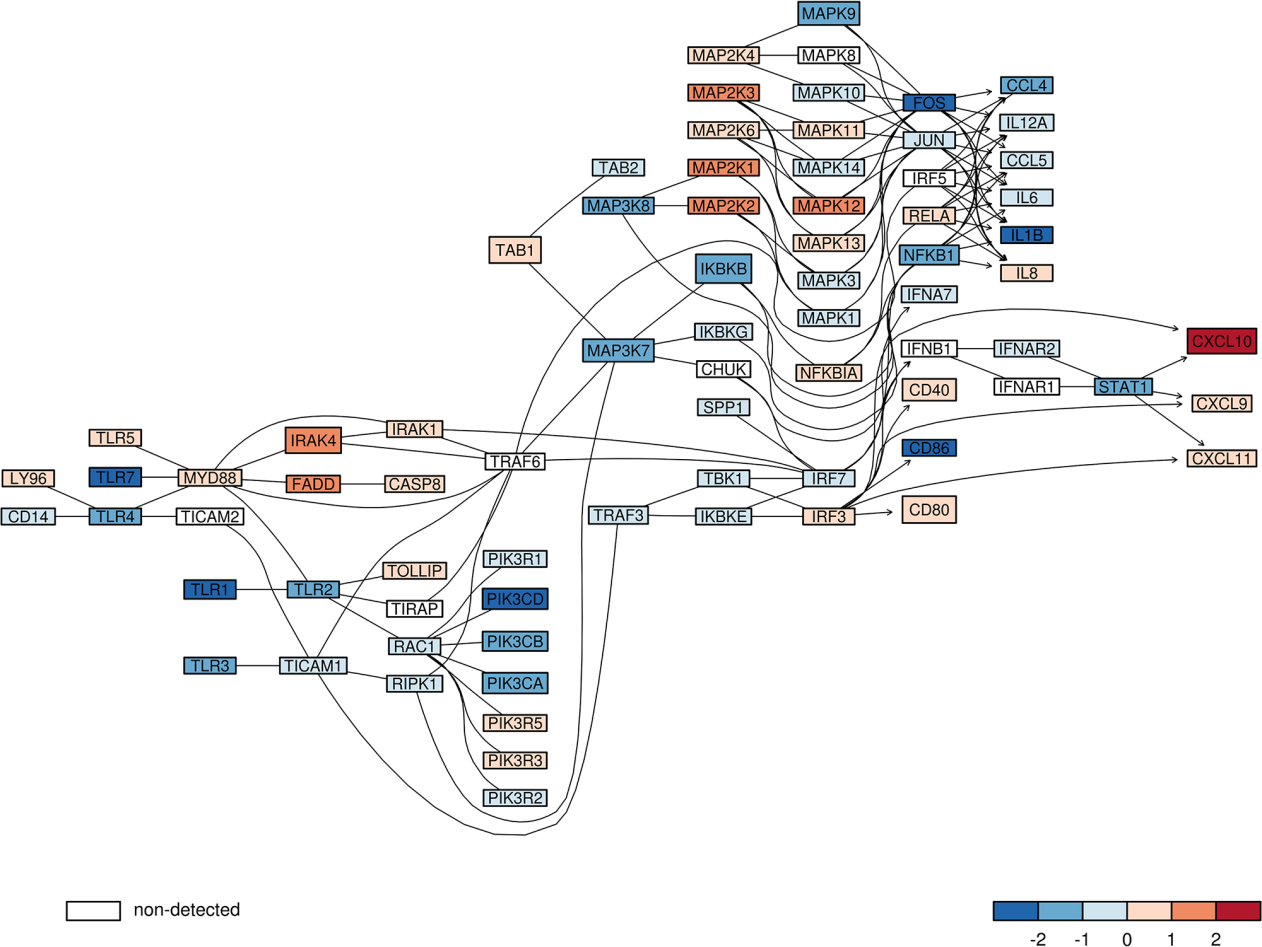
The expression of genes from TLR signaling pathway in low and high grade gliomas The expression of components of the Toll like receptor (TLR) signaling pathway in merged glioma datasets was plotted. The red color marks higher expression in high grade gliomas, while the blue color represents higher expression in low grade gliomas from merged datasets. The color scale represents magnitude of change. A gene which was not measured on analyzed microarrays, but is important for a graph structure, is marked in white. The lines link components of TLR pathway based on known gene-gene relations and their arrows indicate direction of downstream events.

Using signaling pathway definitions from KEGG database [34] we tested whether the “immune” signature is linked to any specific signaling pathway. We found that this “immune” signature was enriched in genes from the Toll-like receptor (TLR) signaling pathway (*p*-value = 0.0003, Fisher's exact test). Almost 20% of genes from the “immune” signature (*CXCL10, TLR7, TLR1, IRAK4, MAPK9, TLR4, CD86, IKBKB*) belong to TLR signaling pathway. We found the reduced expression of many components of this signaling pathway in malignant gliomas in a merged glioma dataset. Many genes coding for Toll-like receptors (TLR 1,2,3,4,7), signaling molecules: PI3KA, B, C, IKKβ, MAPK3K 7, 8, transcription factors FOS, NFκB1 and STAT1, and finally inflammation effectors such as IL-1β, IL-6, CCL4 and 5 were 4- to 8-fold down-regulated in high grade gliomas in comparison to low grade tumors (Fig. 3).

### The expression of the *IKBKB gene* and its protein IKKβ is down-regulated in GBM and GBM derived CD11b^+^ cells

**Figure 4:**
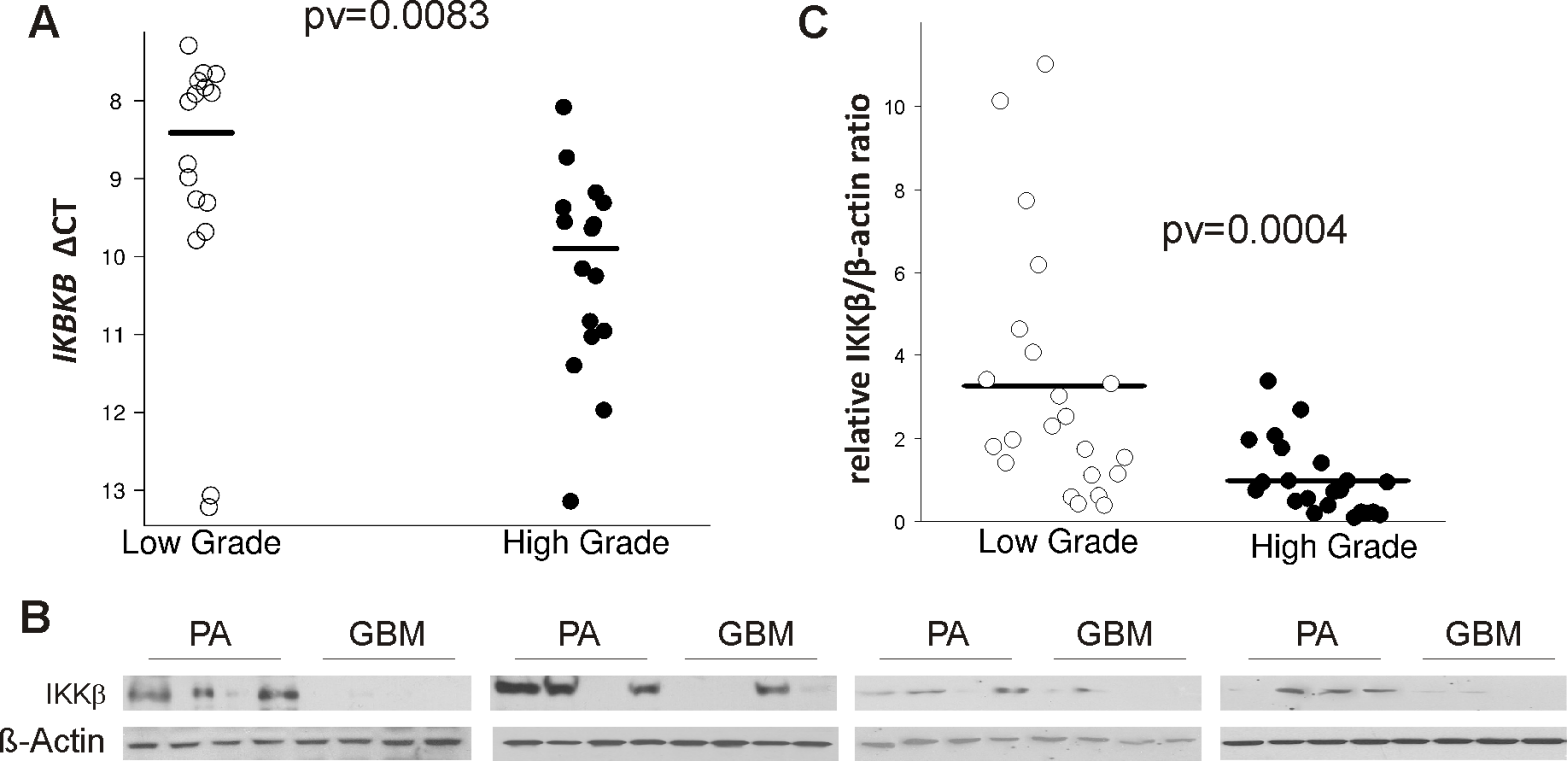
The level of IKBKB mRNA and its protein IKKβ in low and high grade gliomas **A.** Total RNA and proteins were isolated from the same tumor samples after TRIzol procedure. The level of *IKBKB* mRNA in juvenile PA and GBM was determined by qPCR with specific TaqMan probes. Lower ΔCT is consistent with higher gene expression. **B.** The IKKβ level in protein extracts obtained from JPA and in GBM (16 samples/group). After stripping the immunoblots were re-probed with anti-β-actin antibody to ensure equal protein loading. **C.** Densitometric analysis of the IKKβ immunoblots (21 samples/group) has been performed and the results are shown as IKKβ/β-actin ratio in a given sample.

TLR stimulation leads to activation of the nuclear factor kappa B (NFκB) by release of NFκB from the inhibitor of kappaB (IκB) which stimulates the expression of many pro-inflammatory cytokines [35]. IκB is phosphorylated by two kinases IKKα and IKKβ, which bind a regulatory subunit IKKγ (NEMO) [36]. The analysis of merged microarray datasets showed the significantly reduced expression of *IKBKB (*the gene coding for IKKβ) in high grade gliomas. We determined the expression of *IKBKB* by quantitative PCR in independently collected samples from JPA and GBM. JPA is a glial neoplasm with benign behavior and a high survival rate (94% at 10 years) that is attributed to up-regulation of immune defense-associated genes [37]. The level of *IKBKB* mRNA was significantly higher in JPA than in GBM (Fig. 4A). The expression of the IKKβ protein was determined in the same tissues. Immunoblot demonstrates a very low level of the IKKβ protein in almost all GBM samples (15/16), while the expression of IKKβ was high in a majority of JPA (12/16) (Fig. 4B). Densitometric analysis of the immunoblots confirmed the higher expression of IKKβ in JPA than in GBM (Fig. 4C).

**Figure 5:**
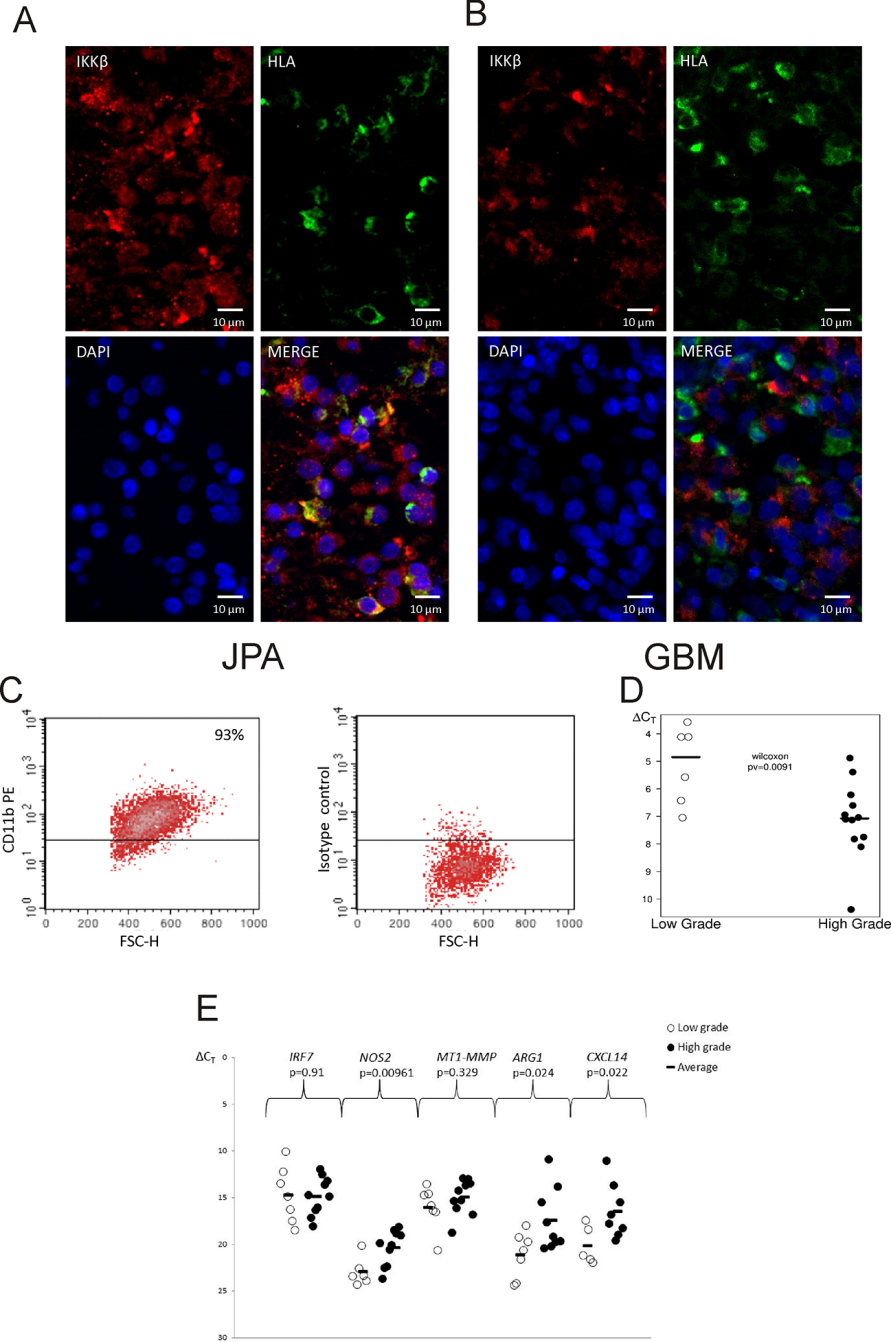
IKKβ expression in microglia/macrophages in low and high grade gliomas **A, B.** Images of double staining for CD11b^+^ (red) and IKKβ (brown) performed on sections of GBM (A) and JPA (B); scale bars 100 μm (insets: 50 μm). **C.** Microglia/macrophages were separated from freshly resected tumors using a magnetic-bead-conjugated anti-CD11b antibody. Purity of the positive fraction stained with anti-CD11b-PE antibody after magnetic separation was 93%. **D–E.** Quantification of the *IKBKB* (D) and M1/M2 marker gene expression (E) in CD11b^+^ cells isolated from low and high grade gliomas. Lower ΔCT is consistent with higher gene expression. Statistical analysis was done with the Wilcoxon testand *t* test, respectively.

The IKKβ immunoreactivity was low in GBM specimens and normal brain in comparison to JPA. (supplementary Fig.2). Double staining of glioma sections with anti-IKKβ (red) and anti-HLA antibodies (green) showed a strong expression of the IKKβ protein in microglia/macrophages (HLA positive cells) in JPA, while the IKKβ immunoreactivity was low in those cells in GBM specimens (Fig. 5A, 5B).

We used the anti-CD11b antibody linked to magnetic beads to isolate CD11b^+^ cells from operative samples of newly diagnosed astrocytoma grade I and II and GBM. Representative histograms demonstrate the high efficacy of cell separation and high purity of the CD11b^+^ fraction (93%) (Fig. 5C). The significantly higher *IKBKB* expression was detected in tumor infiltrating CD11b^+^ cells isolated from low grade tumors than GBM (Fig. 5D). To define a phenotype of glioma infiltrating CD11b+ cells, the expression of genes characteristics for M1 and M2 phenotype [20, 26] was determined. The increased expression of *ARG1, CXCL14* in CD11b+ from GBM may indicate polarization of those cells to the pro-tumorigenic, M2 phenotype (Figure 5E).

**Figure 6:**
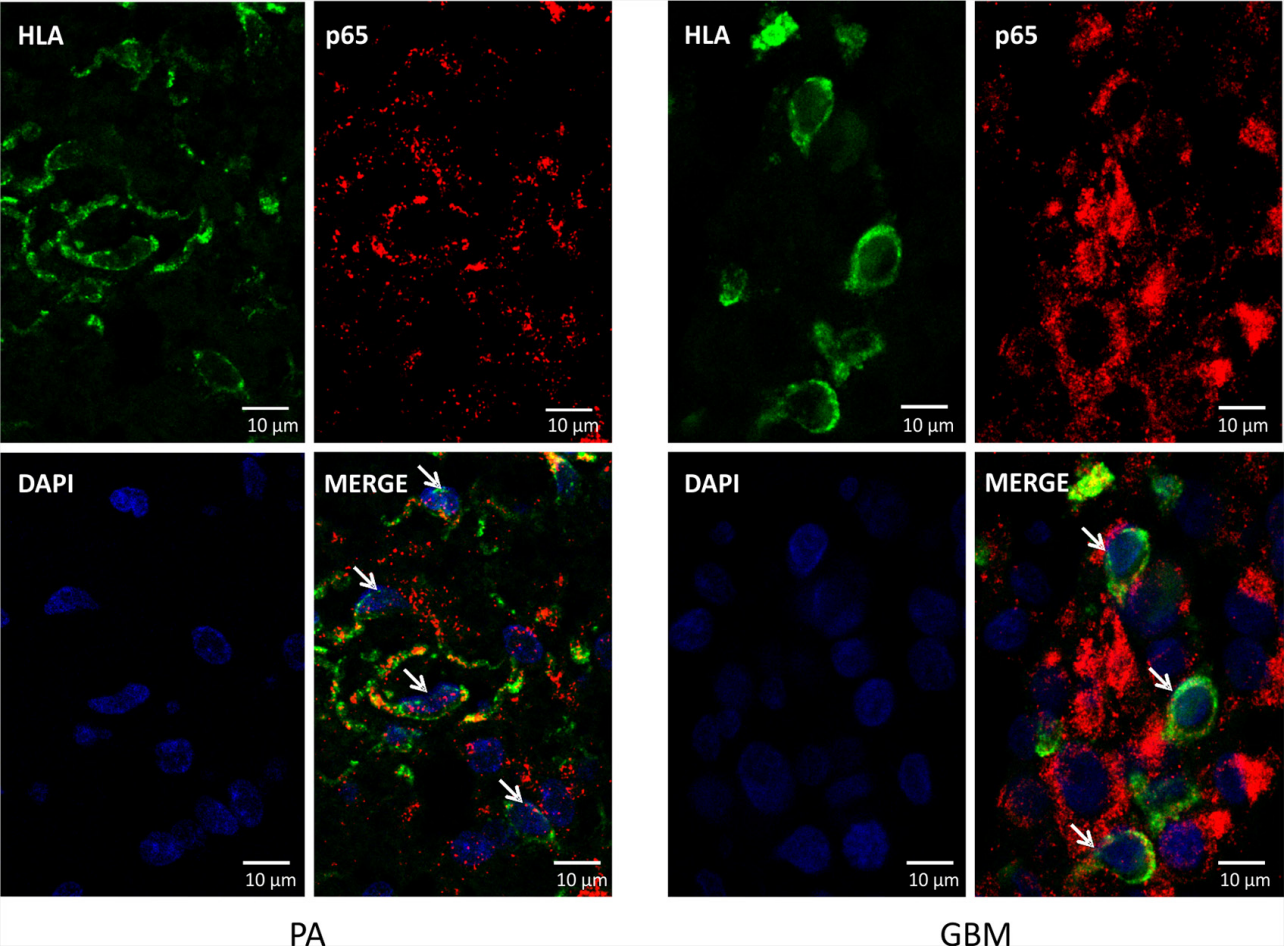
Localization of the nuclear p65 NFκB in glioma-infiltrating microglia/macrophages Double immunofluorescence staining of the p65 NFκB and HLA followed by staining with DAPI was performed on sections from GBM (right) and JPA (left) (*n* = 3/group). Cell nuclei with nuclear localization of p65 NFκB (visualized with confocal microscopy) are indicated by arrowheads.

To evaluate NFκB activation, we analyzed localization of p65 subunit of the NFκB in GAMs using confocal microscopy. Double immunofluorescence staining of the p65 NFκB and HLA was followed by staining with DAPI to visualize cell nuclei. HLA-positive cells with nuclear NFκB staining in JPA tissues are indicated by arrows (Fig. 6). The p65 NFκB subunit is cytoplasmic in many HLA-positive cells in GBM tissues. It shows that NFκB activation is suppressed in microglia/macrophages infiltrating GBM.

**Figure 7:**
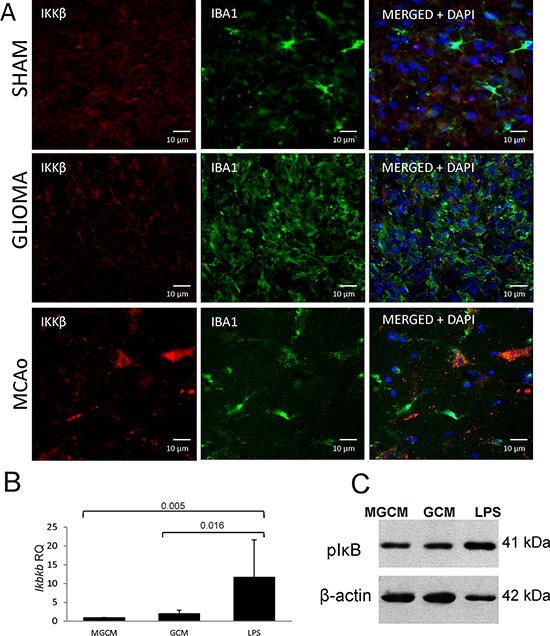
IKKβ is upregulated in inflammatory microglia/macrophages accumulating in the ischemic hemisphere but not in glioma-infiltrating microglia/macrophages **A.** Double immunofluorescence staining of the IKKβ and Iba1 followed by staining with DAPI was performed on sections from sham-operated rats (upper), rats 15 days after C6 glioma cell implantation (middle) and rats 24 h after 90 min ischemia/reperfusion (lower) (*n* = 3/group). The increased IKKβ immunoreactivity is visible in Iba1 positive cells present in the cerebral cortex of rats subjected to transient MCAo. **B.** The *Ikbkb* expression was strongly induced in rat primary microglial cultures 6 h after stimulation with 100 ng/ml lipopolysaccharide (LPS) but not after exposure to glioma conditioned medium (GCM). Representative immunoblot shows the increased level of phospho-IκB 6 h after treatment of microglia with LPS but not in GCM-treated cultures. The increased level of phospho-IκB is indicative of IKKβ signaling activation.

Stimulation of primary cultures of rat microglia with glioma conditioned medium (GCM) induces polarization of those cells into the proinvasive, M2 like cells with the reduced expression of most immune response/inflammatory genes [26]. Fig. 7A shows upregulation of the *Ikbkb* mRNA level in microglia stimulated with lipopolysaccharide (LPS) but not with GCM. Our previous study showed a lack of NFκB activation under such conditions [26]. The IKKβ protein presence in microglia/macrophages was studied in an animal model of stroke (MCAo, middle cerebral artery occlusion) with a pro-inflammatory activation and after implantation of rat C6 glioma cells to an immunocompetent rat where an alternative activation of microglia/macrophages occurs. Double staining of the glioma-bearing and ischemic brain sections with anti-IKKβ (red) and anti-Iba1 antibodies (green) showed a strong expression of the IKKβ protein in microglia/macrophages in the ischemic brain in comparison to sham-operated animals. The IKKβ protein was not detected in Iba1+ cells in glioma-bearing brains (Fig. 7). These results demonstrate the reduced expression of IKKβ protein in microglia/macrophages in experimental gliomas.

## DISCUSSION

Myeloid cells infiltrating malignant gliomas, in particular microglia/macrophages, are characterized by the expression of markers of the pro-tumorigenic, M2 phenotype [11, 12, 38] and impairments of immune responses which results in defective anti-tumor immunity in GBM patients [14, 15]. Experimental studies in microglia-glioma co-cultures, organotypic brain slices and in animal models of gliomas confirmed the pro-invasive and immunosuppressive phenotype of GAMs [19, 21, 22, 25]. Our results corroborate previous studies which have demonstrated accumulation of microglia/macrophages, reflected by moderate or high HLA immunoreactivity in astroglial tumors [9, 10, 13]. Abundance of microglia/macrophages correlated with a tumor severity among adult glioma patients. Notably, morphology of HLA-immunopositive cells was different, with predominance of hypertropic, ramified cells in low grade astrocytomas and mostly amoeboid, fully activated cells in GBM. The meta-analysis of transcriptomic data indicated that while a majority of immune/inflammatory response genes were effectively up-regulated in low grade gliomas, most of these genes were not induced or were down-regulated in high grade gliomas (Fig. 2). Prevalence of down-regulation of immune response genes in high grade gliomas indicates that immunosuppression is widespread, and not restricted to a functional subset of genes. Accuracy of analysis was verified by detection of increased immune/inflammatory gene expression in Parkinson disease which is associated with augmented microglial inflammatory responses [39]. Upregulated expression of the M2 phenotype markers such as *ARG1, CXCL14* in CD11b^+^ cells sorted from GBM, may indicate acquisition of the M2 phenotype.

We computed the “immune” signature based on expression of genes coding for proteins engaged in immune/inflammatory responses, which was validated on the independent set of samples. The “immune” signature separated pilocytic astrocytomas with benign behavior and a highest survival rate attributed to up-regulation of immune defense-associated genes from GBMs. Our conclusion is in line with the results of an analysis of allergy- and inflammation-related genes in GBM tissues which showed a negative association between allergy (characterized by excessive immune responses) and GBM aggressiveness or progression [40]. Increased expression of immune response-related genes correlated with longer survival in the adult mesenchymal GBM and pediatric high-grade gliomas [40, 41]. The immune function and cell type (T cell- and myeloid lineage) genes were enriched in high grade astrocytoma from long-term survivors [32].

Computational analysis of immune response and TLR signaling genes led to our main finding of the reduced expression of *IKBKB* (a gene coding for IKKβ) in high grade gliomas. Down-regulation of the *IKBKB* expression at the mRNA and protein levels in GBM compared to low grade gliomas has been confirmed in an independent set of tumors. Double staining for IKKβ and CD11b^+^ revealed that IKKβ is highly expressed in microglia/macrophages in low grade gliomas but not in GBM. Consistently, we found the higher *IKBKB* expression in CD11b^+^ cells isolated from JPA compared to those from GBM. The expression of *TLR1* and *4* was similar in glioma-derived CD11b^+^ cells and the *TRL7* mRNA was up-regulated in GBM (not shown), therefore there was no defect in upstream TLR signaling. The activation of IKK complex (consisting of IKKα, IKKβ, and NEMO kinases) in response to TLRs, IL-1R, or TNFR stimulation, activates IκB phosphorylation, ubiquitination and degradation, leading to NFκB activation [42]. NFκB transcription factors are essential regulators of inflammatory and immune responses. IKKs participate in activation of interferon response factor (IRF) 3 and 7, induction of type I interferons and the host defense system [36]. The increased expression of *Ikbkb* mRNA and the increase of phospho-IκB level were observed in primary microglial cultures stimulated with LPS but not glioma conditioned medium. Furthermore, a strong upregulation of the IKKβ protein in microglia/macrophages in the ischemic brain but not in glioma-bearing brains supports our clinical observations of defective expression of IKKβ protein in microglia/macrophages exposed to glioma.

Conditional deletion of myeloid IKKβ resulted in resistance to infection [43], attenuated macrophage inflammatory responses, decreased atherosclerotic lesion inflammation, and reduced pro-inflammatory gene expression in primary microglial cultures and during kainate-induced neurodegeneration [44]. This suggests IKKβ participation in inflammatory macrophage activation. Studies in mouse colon and liver cancer models pointed to contribution of NFκB activation in cancer-associated inflammation [45, 46]. Deleting IKKβ in myeloid cells diminished expression of IL-10, IL-12p70, TNF-α cytokines and reduced size of tumors formed by ovarian cancer ID8 cells [45]. The expression of a dominant mutant IKKβ^DN^ or targeted deletion of IKKβ in bone marrow derived or tumor-associated macrophages prevented those cells from enhancing tumor cell invasiveness *in vitro*, enhanced tumoricidal activity and IL-12-dependent NK cell recruitment. These results point to participation of myeloid IKKβ in M2 activation of myeloid cells infiltrating tumors. However, a recent data shows that mice with myeloid-specific IKKβ loss exhibit more rapid growth of cutaneous and lung melanoma tumors due to reduced recruitment of myeloid cells into the tumor, reduced expression of MHCII, and enhanced production of the CCL11 chemokine (negatively regulating dendritic cell maturation) and impaired CD8+T cytotoxicity. Conversely, mice with continuous IKKβ signaling in myeloid-lineage cells exhibited enhanced anti-tumor immunity and reduced B16 melanoma tumor growth [47]. Our results indicate dual roles of glioma infiltrating microglia/macrophages. While the immune response predominates in low grade gliomas, the immunosuppressive and pro-tumorigenic activation occurs in GBM. The reduced level of IKKβ in GAMs from GBM correlates with the impaired immune/inflammatory responses, known to be dependent on NFκB and IRF signaling. Down-regulation of IKKβ could be a limiting factor for initiation of transcriptional responses relaying on NFκB and IRFs. Lack of NFκB activation and interferon-dependent Stat1 phosphorylation was demonstrated in microglial cultures exposed to rat C6 glioma [26]. Our results uncover new mechanisms by which defective IKKβ/NFκB signaling in myeloid cells affects anti-tumor immunity and facilitate glioblastoma progression.

## MATERIALS AND METHODS

### Ethical treatment of research subjects and patient consent

This study was conducted under protocol #14/KBE/2012, which was approved by the Committee of Bioethics of the Children's Memorial Health Institute and the institutional review board of The Institute of Psychiatry and Neurology, Warsaw. Each patient provided a written consent for use of tumor tissues.

### Immunohistochemical and immunofluorescence staining

Tumors included: GI (12 JPA- juvenile pilocytic astrocytoma, 3 subependymal giant cell astrocytoma, 1 ganglioglioma), GII (1 pleomorphic xanthoastrocytoma, 1 pilomyxoid astrocytoma, 2 diffuse astrocytoma, 1 oligodendroglioma), GIII (4 anaplastic astrocytoma, 1 anaplastic ganglioglioma, 2 anaplastic oligodendroglioma), GIV tumors (15 GBM, 3 supratentorial primitive neuroectodermal tumors, 3 anaplastic meningioma). Staining was performed on 5-μm paraffin-embedded tissue sections. Sections were deparaffinized in 60°C for 2 h followed by incubation in xylene, ethanol (100, 90, 70%) and rehydration. Epitopes were retrieved by microwave boiling in pH 6.0 citrate buffer for 20 min.

For microglia/macrophage detection HLA- DP,-DQ,-DR immunoreactivity (shortly HLA) was evaluated [10, 48]. Staining for IKKβ was performed on JPA, GBM and normal brain sections. Endogenous peroxidase was blocked in 0.3% H_2_O_2_ in methanol for 30 min followed by blocking with 1% swine/5% horse serum or 1% swine/5% goat serum. Sections were incubated overnight at 4°C with mouse anti HLA (CR3/43) antibody (DakoCytomation, Glostrup Denmark, dilution 1:200, 3% horse serum) or rabbit anti-IKKβ antibody (Bioworld Technology, St. Louis Park, MN, USA dilution 1:100 in 3% donkey serum) respectively. Next, washed in PBS, incubated with a biotinylated horse anti-mouse immunoglobulin (50 pg/ml PBS) or goat anti-rabbit biotinylated IgG (Vector, Labs., Burlingame, CA, USA; diluted 1:300 in 3% goat serum), then with avidin-DH-biotinylated-HP (horseradish peroxidase) (90 μg/ml PBS) (Vector Labs., Burlingame, CA, USA) for 60 min and with 3,3′-diaminobenzidine (DAB). Sections were stained hematoxylin (Sigma-Aldrich, Munich, Germany), dehydrated through ethanol, cleared in xylene and mounted. Each specimen stained for HLA was scored by two investigators: low immunoreactivity score means no reaction or a weak positive reaction; high immunoreactivity score represents a moderate or strong positive staining. Intensity of staining in relation to the tumor grade was determined with Fisher's Exact test.

Double staining for IKKβ, NFκB p65 and microglial markers (HLA, CD11b or Iba1) was performed. After blocking with 10% donkey serum by 1 h, sections were incubated 48 h at 4°C with rabbit anti-IKKβ antibody (Bioworld Technology, St. Louis Park, MN, USA dilution 1:100 in 3% donkey serum) followed by incubation for 1 h at room temperature (RT) with donkey anti-rabbit AlexaFluor 555 secondary antibody (Life Technologies, Karlsruhe Germany, 1:1000). Sections were incubated overnight at 4°C with mouse anti-HLA antibody (DakoCytomation, Glostrup, Denmark, 1:200 in 3% donkey serum) and next for 1 h in RT with donkey anti-mouse AlexaFluor 488 secondary antibodies (Life Technologies, Karlsruhe Germany, diluted 1:1000) followed by DAPI. For p65 subunit of NFκB or CD11b and HLA staining sections were incubated overnight at 4°C with rabbit anti-NFκB p65 (eBioscience, San Diego, CA USA, diluted 1:200 in donkey serum) or rabbit anti-CD11b (Abcam, Cambridge, UK; 1:100 in 3% donkey serum) and anti-HLA antibodies (DakoCytomation, Glostrup, Denmark, 1:200 in 3% donkey serum). Sections were next incubated for 1 h in RT with donkey anti-mouse AlexaFluor 488 and donkey anti-rabbit AlexaFluor 555 secondary antibodies (Life Technologies, Karlsruhe Germany, diluted 1:1000) followed by DAPI (1:1000). Sections were mounted with a fluorescent mounting medium (DakoCytomation, Glostrup, Denmark) and visualized with a Confocal Leica SP8 Microscope or Leica DFC 300FX. Double staining for IKKβ and Iba1 was performed by incubating sections with rabbit anti-IKKβ antibody (Bioworld Technology, St. Louis Park, MN, USA dilution 1:100 in 3% donkey serum) and goat anti-Iba1 antibody (Novus Biologicals, Littleton, USA) for 24 h at 4°C followed by incubation for 1 h at room temperature with donkey anti-rabbit AlexaFluor 555 secondary antibody and donkey anti-goat AlexaFluor 488 (Life Technologies, Karlsruhe Germany, 1:1000). In control experiments, primary antibodies were omitted. Sections were mounted with fluorescent mounting medium and visualized with a Nikon Eclipse 80i. Images were captured using Image Pro-Plus 5.0 software.

### Microarray meta-analysis

Raw microarray data from five Affymetrix U133A/plus2 glioma datasets were downloaded from NCBI GEO: GSE5675 [49] (41 profiles of JPA), GSE2817 [50] (10 profiles of high grade and 16 profiles of low grade gliomas), GSE8692 [51] (9 profiles of high grade gliomas, 3 profiles of low grade gliomas), GSE4271 [52] (100 profiles of GBM) or ArrayExpress: E-MTAB-1928 [53] (13 profiles of high grade gliomas).

All data computation were performed using R and relevant Bioconductor software. Quality of the downloaded CEL files (determined using affyQCReport Bioconductor package) was acceptable, only a few files did not meet the basic quality criteria (GAPDH 3′:5′ ratio 1.25, - actin 3′:5′ ratio 3 and a scale factor within 3-fold of the mean of all chips) and were excluded. Microarray datasets were preprocessed with GC-RMA separately. To remove unreliably measured genes and to uniquely map probe sets to genes, we applied filtering and mapping procedures [33]. We applied: Present/Absent/Marginal filtration to verify specificity of measured signal; and transformation of probe set measurements into gene measurements, excluding probe sets annotated to more than one gene (annotations were from the Ensembl 66). Only genes that passed filtration in all experiments were used in analyses. To allow comparison between different data sets, we applied quantile normalization to the merged dataset and removed dataset-specific bias. The association between original data sets (before merging) is clearly visible (suppl.fig.1).

To establish correlation between malignancy grade and gene expression profiles Fisher's exact test was employed. First, we used a one-sided *t*-test (under the null hypothesis that expression of each tested gene is higher in high grade gliomas) and obtained a list of differentially expressed genes (*pv* < 0.01). We selected genes from particular Gene Ontology (GO) term using GOstats Bioconductor package. Next, we tested whether a given GO term is overrepresented in the list of differentially expressed genes using Fisher's exact test for independence in a 2 × 2 (contingency) table. Methodology was verified by comparing similarly preprocessed expression data of Parkinson Disease and controls (GSE7621).

GO terms: Immune Response (GO:0006955), Innate Immune Response (GO:0045087) and Inflammatory Response (GO:0006954) were used to analyze the immune gene expression in gliomas. To find a set of genes (an “immune” signature) which best discriminates between benign and malignant gliomas, we applied the Prediction Analysis of Microarrays method [29]. An independent dataset (E-MTAB-1928) of 13 GBM and 11 JPA generated in our laboratory was used for validation. Definition of Toll-like receptor signaling pathway was downloaded from KEGG - Kyoto Encyclopedia Genes and Genomes (version 62) [34].

### RNA isolation and global gene expression profiling

Snap-frozen 13 GBM and 11 JPA were obtained from the Canadian Brain Tumor Tissue Bank (London Health Sciences Centre, Ontario CA) and the Children's Memorial Health Institute (Warsaw, Poland). Total RNA was prepared using Tri-Reagent extraction (Sigma-Aldrich, Munich, Germany) followed by the RNeasy Mini Kit (Qiagen, Hilden, Germany) isolation as described [23]. RNA quality/yield was verified by Bioanalyzer 2100 (Agilent Technologies, Santa Clara, CA) and NanoDrop 2000 (Thermo Scientific, München Germany). Samples were analyzed on Human HT-12 Expression BeadChips at the Cambridge Genomic Core Facility, UK. Array data processing and analysis were performed using IlluminaGenomeStudio software and normalized using the R Bioconductor beadarray and limma packages as described. Probes not detected (*P* value > 0.01) on the microarrays were removed from further analysis.

### Preparation of protein extracts and western blot analysis

Protein extracts were prepared from pellets obtained after isolation of RNA. Pellets were dissolved in 8 M Urea, 50 mM CHAPS, 20 mM DTT for 3 h in 75°C. Protein extracts were prepared by adding 5 × Laemmli sample buffer, heating for 15 min. Proteins were separated by SDS-PAGE and transferred onto nitrocellulose membranes (Amersham, Uppsala, Sweden) as described [21]. For Western blot analysis a polyclonal anti-IKKβ antibody and HP-conjugated anti-rabbit IgG (Cell Signaling Technology, Beverly, MA, USA) were used. The membranes were stripped and re-probed with HP-conjugated anti-β-Actin antibody (Sigma-Aldrich, Saint Louis, MO, USA). Densitometric analysis was performed using ImageJ.

### Quantification of gene expression

cDNA was synthesized by extension of the oligo(dT)_15_ primers (2.5 mmol/L) using 200 units of M-MLV reverse transcriptase (Sigma-Aldrich, Germany). Reaction volume (10 μl) consisted of cDNA equivalent to 18.75 ng RNA, TaqMan Fast Universal PCR Master Mix 2x (Applied Biosystems, Darmstadt, Germany) and *IKBKB* probe (Hs00826074_m1, Applied Biosystems, Darmstadt, Germany), the primer set for *CXCL14* expression (Qiagen NM_004887) or specific primers (Supplementary Table 3). *GAPDH* (Hs02758991_g1, Applied Biosystems, Darmstadt, Germany) was used as an internal reference gene. Amplification conditions were as follows: 50°C for 2 min, 95°C for 10 min, by 40 cycles of 15 s at 95°C and 1 min. at 60°C. Relative quantification of gene expression was determined using the comparative CT method. The reference brain RNA was a mixture e of 23 normal brains (Ambion, Austin,TX, USA).

### Immunomagnetic isolation of CD11b-positive cells and gene expression quantification

Fresh tumor samples were collected in cold HBSS (Hank's Balanced Sodium Solution without Ca^2+^ and Mg^2+^) with antibiotics (50 U/ml penicillin, 50 μg/ml streptomycin) and processed within 24 h. The tissue was mechanically and enzymatically dissociated with the gentle MACS*™*Dissociator and Neural Tissue Dissociation Kit (MiltenyiBiotec, Cologne, Germany). Cells were labeled with CD11b MicroBeads, loaded onto MACS Columns placed in the magnetic field of MACS Separator. CD11b^+^ cells were eluted as a positive fraction, stained with CD11b-PE antibody (BD Pharmingen, San Diego California) and checked for purity using flow cytometry (FACSCalibur, BD Bioscences, NJ, USA) and CellQuest software. Total RNA from sorted CD11b^+^ cells was isolated with RNeasy Mini Kit (Qiagen, Hilden, Germany). Relative quantification of gene expression was determined using the comparative CT method.

### Microglial cultures and animal experiments

The use of microglial primary cultures and all animal experimentation have been approved by the Local Ethics Committee (#582/2014). Microglial cultures were prepared from cerebral cortices of 1-day-old Wistar rat pups as previously described [26]. Cells were plated at a density of 3 × 10^5^ cells/cm^2^ on poly-L-lysine-coated flasks in Dulbecco's modified Eagle medium (with Glutamax, 4.5 g/L glucose, 10% FBS (Gibco), 100 U/mL penicillin, and 0.1 mg/mL streptomycin). To induce M1 or M2 activation microglial cultures were stimulated with 100 ng/ml lipopolysaccharide (Sigma) or glioma conditioned medium for 6 h, then washed and processed for RNA and protein isolation as previously described [26].

All animal experiments were performed in accordance with national guidelines, current European Union (Directive 2010/63/EU) by authorized investigators. C6 glioma cells (5 × 10^4^ cells/in 2.5 μl of DMEM) were implanted into the right striatum of 250 mg male Wistar rats. For focal brain ischemia, transient middle cerebral artery occlusion (MCAO) was evoked by filament occlusion of the right MCA as previously described [54]. Rats (250 mg) were anesthetized with isoflurane in oxygen-enriched air by facemask and unilateral MCAO was performed by inserting a 7–0 nylon monofilament into the carotid artery. MCA was occluded for 90 min followed by reperfusion. Sham-operated animals underwent the same procedure, except of the introduction of the filament. At day 15^th^ after glioma cell implantation or 24 h after MCAo animals were euthanized and intracardially perfused with 4% paraformaldehyde. Brains were removed, frozen with dry CO_2_ and serial coronal sections were collected.

## SUPPLEMENTARY FIGURES AND TABLES


