# Supplementary material for: Down-regulation of IKKβ expression in glioma-infiltrating microglia/macrophages is associated with defective inflammatory/immune gene responses in glioblastoma
Source: Oncotarget. 2015 Sep 29;6(32):33077–90. doi: 10.18632/oncotarget.5310 (PMC4741750; doi:10.18632/oncotarget.5310)
Supplement: Supplementary file 1 [file oncotarget-06-33077-s001.pdf]

SUPPLEMENTARY FIGURES AND TABLES

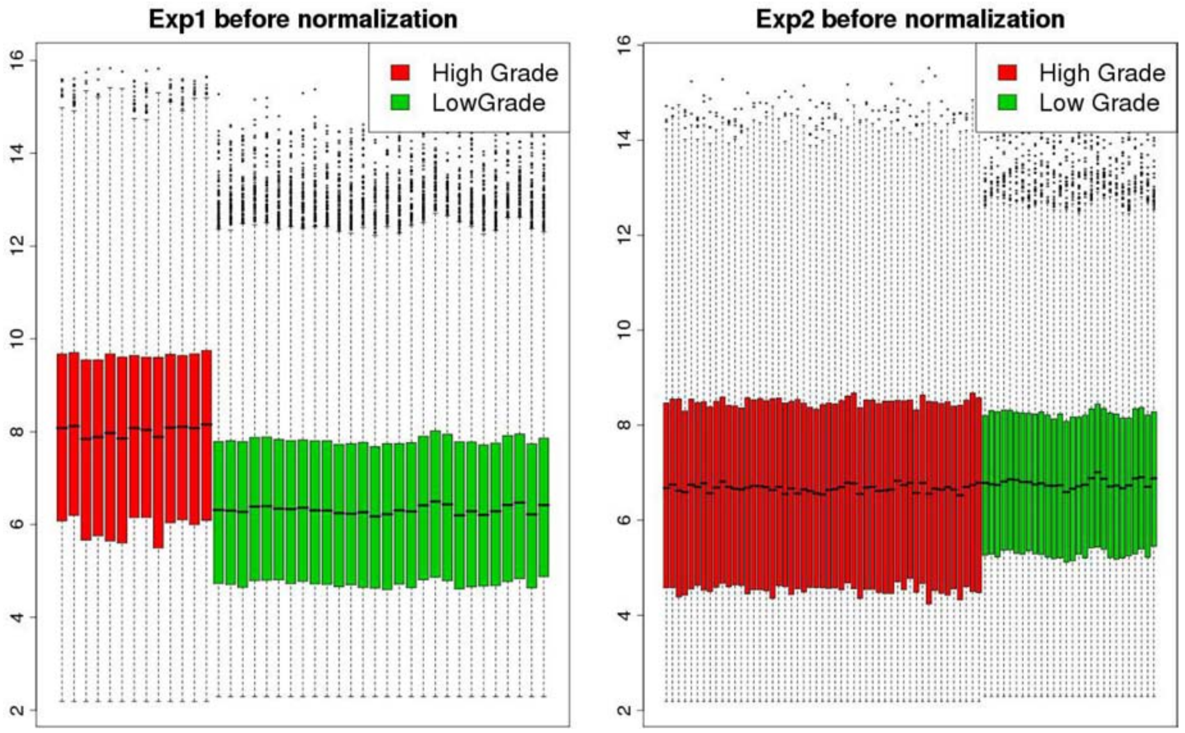

Supplementary Figure S1: Quantile normalization. The boxplots present overall gene expression distributions in all samples. The association between original data sets (before merging) is clearly visible.

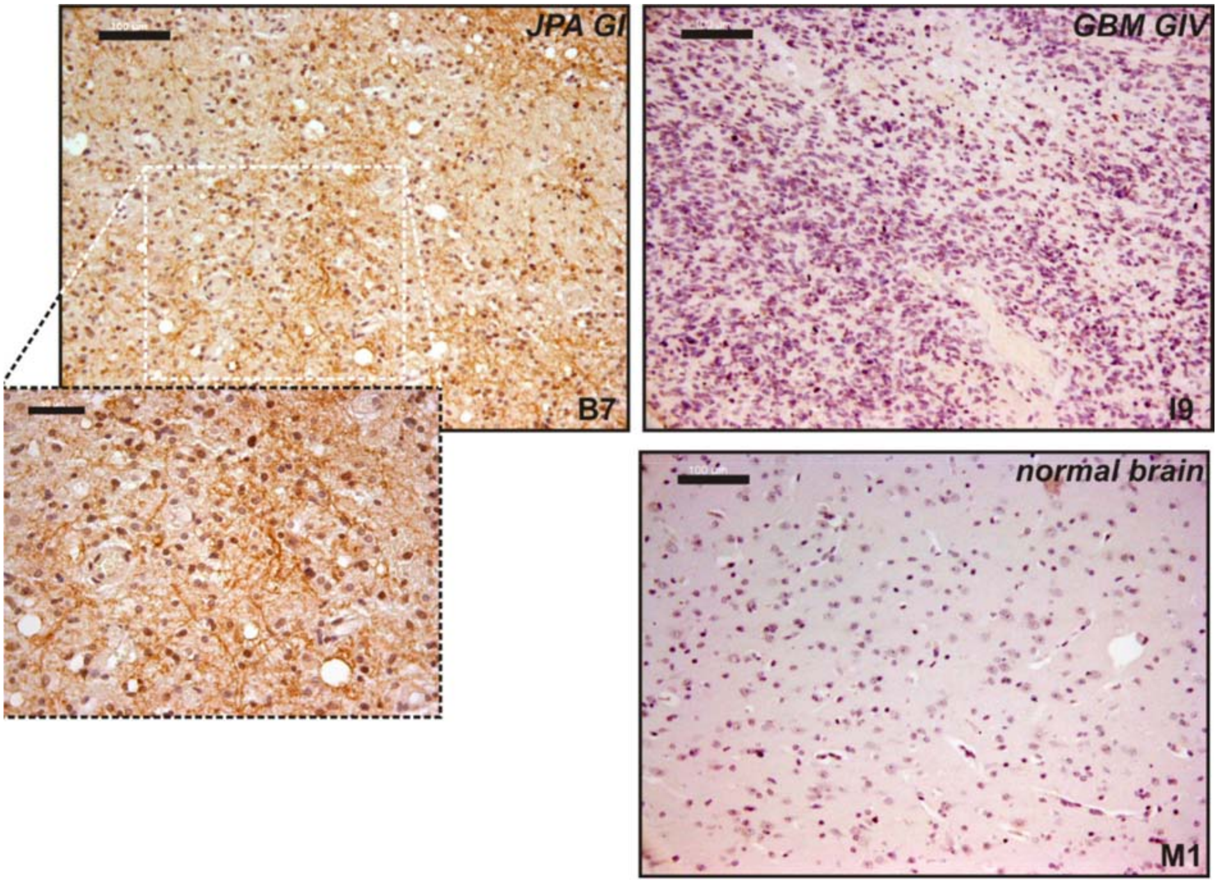

Supplementary Figure S2: Representative images of immunohistochemistry for IKKβ (brown) in JPA, GBM and normal brain.

Supplementary Table S1: Histopathological classification of tumors enclosed in the merged dataset and used for a meta-analysis

Data set name	Gene profile repository	Gene profiles of low grade gliomas	Gene profiles of high grade gliomas	Reference
GSE5675	NCBI GEO	41 juvenile pilocytic astrocytoma WHO grade I		31
GSE2817	NCBI GEO	4 oligodendroglioma WHO grade II, 6 oligoastrocytoma WHO grade II and 6 astrocytoma WHO grade II	6 anaplastic oligoastrocytoma WHO grade III,4 anaplastic astrocytoma WHO grade III	32
GSE8692	NCBI GEO	2 mixed gliomas WHO grade II, 1 oligodendroglioma WHO grade II	6 GBM WHO grade IV, 3 gliosarcomas WHO grade IV	33
GSE4271	NCBI GEO		100 GBM WHO grade IV	34
E-MEXP-1507	ArrayExpress		8 oligodendroglioma WHO grade III and 5 GBM grade IV	35

Supplementary Table S2: Gene names and expression scores in GBM and JPA

	GBM.score	JPA.score
PTPN11	−0,1641	0,3609
HFE	0,1414	−0,3111
IL6ST	−0,1408	0,3097
C7	−0,1393	0,3065
PTPRC	−0,1372	0,3018
TCF7	0,1271	−0,2796
AZGP1	−0,1231	0,2709
DUSP10	0,1161	−0,2555
ITCH	0,1147	−0,2523
CXCL10	0,1078	−0,2372
CDK1	0,1059	−0,233
P2RY14	−0,1012	0,2226
NCAM1	−0,0975	0,2146
CX3CL1	0,0923	−0,203
TLR7	−0,0911	0,2004
TLR1	−0,0748	0,1646
IL27RA	−0,06	0,132
IGF1R	−0,0581	0,1279
KCNJ10	−0,0579	0,1275
ST6GAL1	−0,0565	0,1242
PRDX2	0,0523	−0,1152
ADORA1	0,0508	−0,1118
POLR3F	−0,047	0,1035
MR1	0,0467	−0,1028
CD86	−0,0415	0,0913
MEF2A	−0,041	0,0901
PLCG1	0,0383	−0,0842
SMAD6	−0,0365	0,0803
TLR4	−0,0336	0,0738
IRAK4	0,0331	−0,0727
VCAM1	0,0326	−0,0718
MAPKAPK3	0,0309	−0,068
RPS6KA3	−0,0302	0,0664
ADORA3	−0,0286	0,0629
IL1B	−0,0282	0,0621
MGLL	−0,0274	0,0602

(*Continued*)

	GBM.score	JPA.score
NT5E	−0,0245	0,0539
FADD	0,0227	−0,0499
GNL1	0,0223	−0,049
RGS1	−0,0212	0,0467
IL1R1	−0,0209	0,046
FCGR3B	−0,0204	0,0449
MAPK9	−0,019	0,0419
IL1RAPL1	−0,0186	0,041
LAT2	0,0183	−0,0403
PLEKHA1	−0,0181	0,0397
JAK2	−0,0179	0,0395
OLR1	−0,0178	0,0392
IL16	−0,0169	0,0372
SPN	0,0168	−0,037
CYLD	−0,0155	0,0341
PSTPIP1	−0,0144	0,0316
MEF2C	−0,0139	0,0305
APP	−0,0129	0,0283
SWAP70	−0,0125	0,0276
ECSIT	0,0124	−0,0273
CD37	−0,0123	0,0271
LIG4	−0,0116	0,0256
COLEC12	−0,0114	0,0251
SEMA4D	−0,0099	0,0218
SOCS3	−0,0077	0,017
MAP3K7	−0,0076	0,0167
ICAM2	−0,0061	0,0134
FAS	−0,0054	0,0118
IKBKB	−0,0036	0,0079
CD55	−0,0019	0,0041
TGFB1	−4e-04	0,001
PIK3CB	−3e-04	8e-04

Supplementary Table S3: Primer sequences for qPCR

Gene name	Forward primer	Reverse primer
TLR1	5′TTTTGTGGCCAGGGTCTTCAT 3′	5′ TCCTTTTTGTAGGGGTGCCCA 3′
TLR4	5′CCAAGAACCTGGACTGAGC 3′	5′ TGTCTGGATTTCACACCTGGAT 3′
TLR7	5′ TCAAGAAAGTTGATGCTATTGGGC 3′	5′ ACCATCTAGCCCCAAGGAGT 3′
IRF7	5′GCGGTGCAAGAGCCCAGCCC3′	5′CGTGCAGCTCGGGTGTCCCA3′
NOS2	5′GCAGGACTCACAGCCTTTGGACC3′	5′GGCACGGCTGGATGTCGGACT3′
ARG1	5′TCTCAAAGGGACAGCCACGAGGA 3′	5′TCAGCAAAGGGCAGGTCCCCATA3′
MT1-MMP	5′CTCGGCCCAAAGCAGCAGCT 3′	5′GCGATGGCCGCTGAGAGTGA 3′
